# Supplementary material for: Female genital schistosomiasis burden and risk factors in two endemic areas in Malawi nested in the Morbidity Operational Research for Bilharziasis Implementation Decisions (MORBID) cross-sectional study
Source: PLoS Negl Trop Dis. 2024 May 8;18(5):e0012102. doi: 10.1371/journal.pntd.0012102 (PMC11104661; doi:10.1371/journal.pntd.0012102)
Supplement: S1 Text — (DOCX) [file pntd.0012102.s002.docx]

**S1 text: Sample selection procedure and randomization for the MORBID-FGS sub-study**

A list of girls and women aged 15-65 years old from the main *parent* MORBID study was created. Simple random sampling was then used to determine a random sample of 1,000 girls and women selected for the MORBID-FGS sub-study. This was conducted in STATA and overseen by the project manager (SK).
