# Supplementary material for: Female genital schistosomiasis burden and risk factors in two endemic areas in Malawi nested in the Morbidity Operational Research for Bilharziasis Implementation Decisions (MORBID) cross-sectional study
Source: PLoS Negl Trop Dis. 2024 May 8;18(5):e0012102. doi: 10.1371/journal.pntd.0012102 (PMC11104661; doi:10.1371/journal.pntd.0012102)
Supplement: S2 Text — (DOCX) [file pntd.0012102.s003.docx]

**S2 text: Study design of the Morbidity Operational Research for Bilharzia Implementation Decisions (MORBID) study**

The MORBID study was a cross-sectional community-based study in Malawi to identify *S. haematobium* specific morbidity markers in four age groups (2–6-year-olds, 7–13-year-olds, 14–19-year-olds and adults older than 20) and to identify the level of schistosomiasis infection below which little or no schistosomiasis-specific morbidity can be detected in each age group*.* Participants of the MORBID study were recruited from the Nsanje and Chikwawa districts in Southern Malawi. The unit of evaluation of the study was the village, as programmatic activities are evaluated at the aggregate level. Villages were selected, half from low prevalence areas (0%) and half from high prevalence areas (>25%), as determined by epidemiological mapping reassessment survey data (2017-2019) and ecological factors such as land use, elevation, groundwater, and land surface data from satellite imagery. Across two districts 30 villages were identified which were 0% and 30 villages which were >25% prevalence (60 villages in total).
