# Supplementary material for: Female genital schistosomiasis burden and risk factors in two endemic areas in Malawi nested in the Morbidity Operational Research for Bilharziasis Implementation Decisions (MORBID) cross-sectional study
Source: PLoS Negl Trop Dis. 2024 May 8;18(5):e0012102. doi: 10.1371/journal.pntd.0012102 (PMC11104661; doi:10.1371/journal.pntd.0012102)
Supplement: S3 Text — (DOCX) [file pntd.0012102.s004.docx]

**S3 text: Sample participants and sample size for the *parent* MORBID study**

During the MORBID study, a total of 200 individuals aged over 2 years old were selected in each village using randomized sampling. Fifty participants were sampled from each age groups including pre-school aged children (2-6 years old), school aged children (7-13 years old), adolescents (14-19 years old), and adults (aged over 20 years old). Participants were eligible to participate if they were over 2 years of age, have been resident in the study area for the last 6 months and provided informed written consent. Any participant who was observably ill or had an underlying medical condition, as judged by the study nurse, was excluded from the study.

The main objective of the MORBID-FGS sub-study was to describe the FGS prevalence across different endemicity settings in Malawi by different diagnostic methodologies. The sample size calculation for the MORBID-FGS study was based on the sample size and recruitment from the main MORBID study. The sample size for the MORBID study was calculated to have 80% power to detect schistosomiasis-associated morbidity at a threshold of 7.5% schistosome infection prevalence. To accomplish this, 30 village were randomly selected, half from low prevalence areas (0%) and half from high prevalence areas (>25%) (60 villages in total. Sample size estimates were determined via simulation in R (version 3·5·1) using a segmented regression model in the segmented package. An equal number of villages were allotted to each prevalence group. For the 25-100% group, a Beta distribution with alpha=1 and beta=3 was used in order to generate fewer extremely high prevalence values. Based on the sample size calculation, it was estimated that 50 people had to be selected for each group, resulting in 200 individuals per village. With the above sample size powered to detect schistosomiasis related morbidity, the FGS study was adequately powered.
