# Supplementary material for: Female genital schistosomiasis burden and risk factors in two endemic areas in Malawi nested in the Morbidity Operational Research for Bilharziasis Implementation Decisions (MORBID) cross-sectional study
Source: PLoS Negl Trop Dis. 2024 May 8;18(5):e0012102. doi: 10.1371/journal.pntd.0012102 (PMC11104661; doi:10.1371/journal.pntd.0012102)
Supplement: S4 Text — (DOCX) [file pntd.0012102.s005.docx]

**S4 text: Randomized sampling procedures for the *parent* MORBID study**

1. Selection if the household listing is available

Households for recruitment of participants were selected from a household listing provided by the village lead or administrative leader. Team leaders ensured that the household list provided was fully up to date and captured all households within the area. Every *x* households in the village are sampled with the initial household being a random number between 1 and *x,* where *x* is the sampling fraction as calculated below:

$$\boldsymbol{x=}\frac{\boldsymbol{Total number of households in village}}{\boldsymbol{Number of households to survey}}$$

After calculating *x,* the first household was selected by randomly selecting a number between 1 and *x.* Random number selection was done in the field by writing numbers on pieces of paper, folding them up, placing them in a container and mixing before drawing one out at random, and then selecting the household that is on this row in the village list. Sampling should then proceed in this manner with every *x*^th^ household being sampled.

**Example of selection of households with a village list:**

1. The protocol is to sample 12 households in the village.
2. The village list shows that there are 200 households in the village.
3. Therefore *x* = 200 / 12 = 16·7, which is rounded down to 16
4. The numbers 1 – 16 are written on pieces of paper, folded up and placed in a container and mixed up. The random piece of paper drawn out is 5.
5. The household on the 5^th^ row of the village list is identified.
6. The second household to select for interviews is 5 + 16 = 21. The household on the 21^st^ row of the village list is identified.
7. Sampling then continues to households 37 (= 21 + 16), 53, 69, 85, 101, 117, 133, 149, 165, 181 and 197 giving 12 households sampled in total.

2. Selection if the household listing is NOT available

If an up-to-date household list is not available, then the total number of households were counted and numbered. This was conducted by the Village’s Health Surveillance Assistant, who then instructed the team on how the numbering was conducted. The randomization procedure was then the same as above.

3. Selecting individuals within a household

All eligible individuals in the household were invited to participate in the study. If there were less than 50 individuals in one of the age categories across the village, but sufficient numbers in the other categories, the team was informed to recruit only for the missing age category.

If an individual refused (or their parent/guardian refused) their participation, the team continued to recruit the remaining individuals in that HH. If all refused, follow as per the HH, the household was replaced with the next one in the direction of travel.
