# Supplementary material for: Female genital schistosomiasis burden and risk factors in two endemic areas in Malawi nested in the Morbidity Operational Research for Bilharziasis Implementation Decisions (MORBID) cross-sectional study
Source: PLoS Negl Trop Dis. 2024 May 8;18(5):e0012102. doi: 10.1371/journal.pntd.0012102 (PMC11104661; doi:10.1371/journal.pntd.0012102)
Supplement: S5 Text — (DOCX) [file pntd.0012102.s006.docx]

**S5 text: Diagnosis of urinary *Schistosoma (S.) haematobium* by urine filtration**

The MORBID-FGS sub-study was performed after a short time from the main *parent* MORBID study. Prevalence and intensity of *S. haematobium* infection for all participants, including girls and women later selected for the MORBID-FGS sub study, were assessed using urine filtration diagnostic methods. Morbidity indicators in the MORBID study were assessed by carrying out reagent strip testing urine for hematuria and proteinuria, anthropometry, finger prick testing for prevalence of anemia and malaria, body measurements for prevalence and severity of stunting and wasting and, ultrasonography of the urinary tract to determine bladder wall and urinary tract abnormalities.

Urine filtration was performed in the laboratory in the two study areas by a trained laboratory technician. A single urine sample (~250 ml) was collected for each study participant between 10am and 2pm as part of the main MORBID study. Specimens were examined using urine filtration as soon as possible after collection.

For the urine filtration, after preparing the filter holder by inserting the nucleopore filter, 10ml of urine specimen were drawn into a syringe attached to the filter. The syringe was held in a vertical position and the plunger was pressed down to push all the urine through the filter and in the sample’s container. Excess urine was removed from the syringe by passing air through it. The top side of the filter, where the eggs were captured, was then placed on top of a glass microscope slide. One drop of Lugol’s iodine was added and after 15 seconds, the slide was examined under a microscope at low power (x40). The total number of eggs on the filter was recorded. Slides were read within an hour of the urine sample being taken to ensure that eggs were viable and allow them to become translucent. 10% of slides were randomly selected and re-examined by a more experienced technician for quality control. The discrepancy in egg count was less than 10%.
