# Supplementary material for: Female genital schistosomiasis burden and risk factors in two endemic areas in Malawi nested in the Morbidity Operational Research for Bilharziasis Implementation Decisions (MORBID) cross-sectional study
Source: PLoS Negl Trop Dis. 2024 May 8;18(5):e0012102. doi: 10.1371/journal.pntd.0012102 (PMC11104661; doi:10.1371/journal.pntd.0012102)
Supplement: S6 Text — (DOCX) [file pntd.0012102.s007.docx]

**S6 text: Genital DNA isolation and PCR**

Genital PCR from cervicovaginal swabs was performed at the Clinica Microbiology Laboratory (CML) of the Leiden University Medical Centre (LUMC). After the samples were vortexed, the complete PrimeStore solution was transferred to a 2mL tube containing Precellys Soil grinding beads (Bertin technology, Montigny-le-Bretonneux, France). Following bead-beating, 5 minutes at 2200 rpm (IKA VXR basic Vibrax orbital shake), DNA was extracted using The MagNA Pure 96 DNA and Viral NA small volume kit and Pathogen Universal 200 protocol on the MagNA Pure 96 (Roche Diagnostics, Penzberg, Germany). DNA amplification and detection were performed with the CFX-96 Real Time PCR Detection System (BioRad, California, USA). The CML is EN ISO 15189 accredited and the Schistosoma PCR is included in the scope. Interlaboratory comparison is covered by performing the Helminth External Molecular Quality Assessment Scheme (HEMQAS) provided by the Dutch Foundation for Quality Assessment in Medical Laboratories (SKML) (1).
