# Supplementary figures and images for: Female genital schistosomiasis burden and risk factors in two endemic areas in Malawi nested in the Morbidity Operational Research for Bilharziasis Implementation Decisions (MORBID) cross-sectional study
Source: PLoS Negl Trop Dis. 2024 May 8;18(5):e0012102. doi: 10.1371/journal.pntd.0012102 (PMC11104661; doi:10.1371/journal.pntd.0012102)

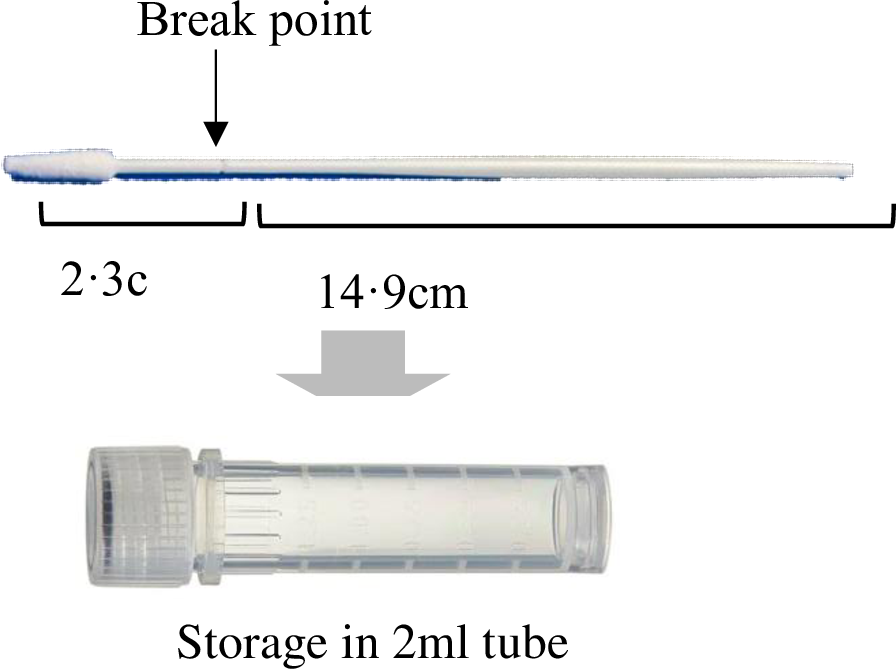

Supplement: S1 Fig — (TIF) [file pntd.0012102.s008.tif]

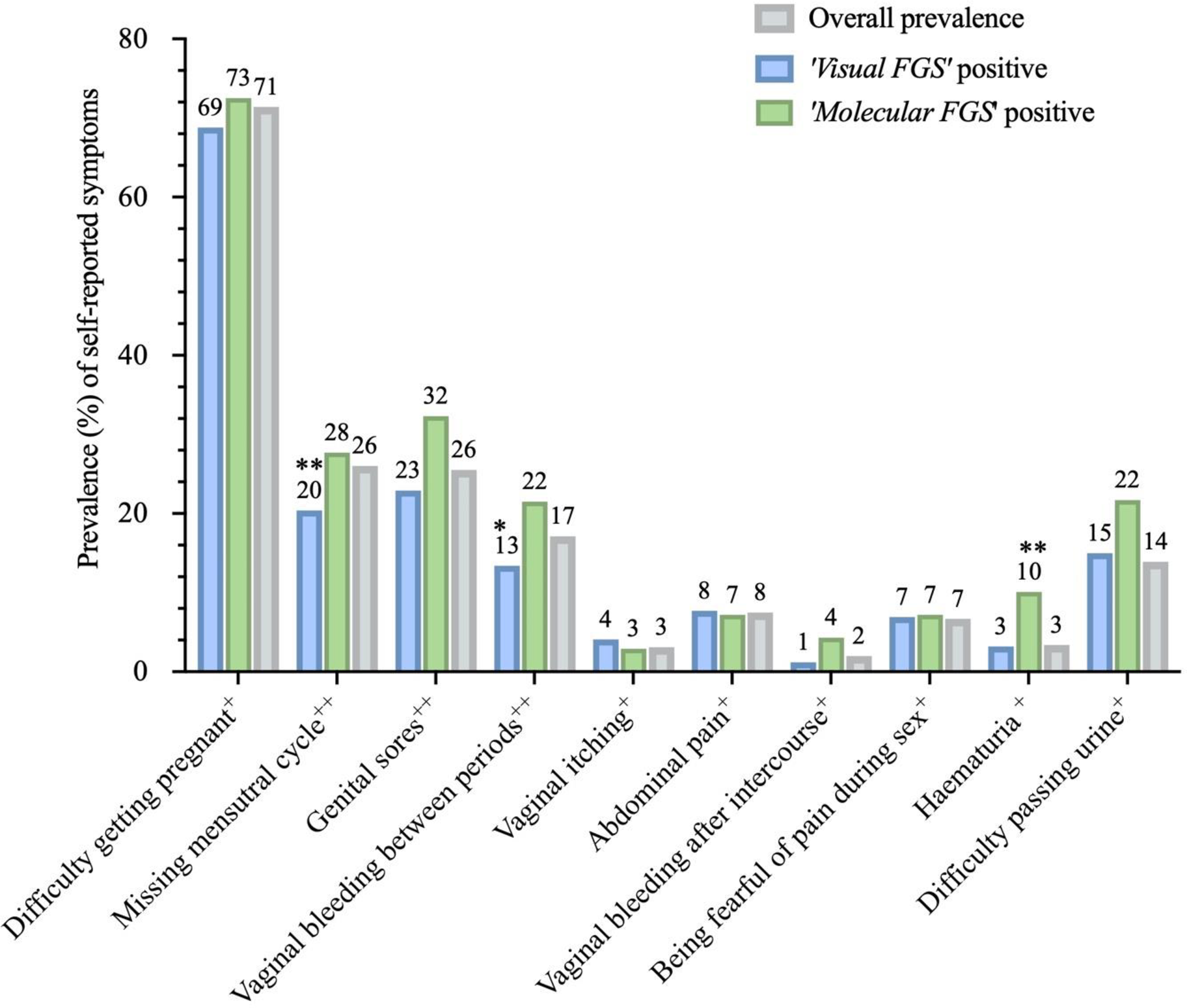

Supplement: S2 Fig — (TIF) [file pntd.0012102.s009.tif]
