# Supplementary material for: Female genital schistosomiasis burden and risk factors in two endemic areas in Malawi nested in the Morbidity Operational Research for Bilharziasis Implementation Decisions (MORBID) cross-sectional study
Source: PLoS Negl Trop Dis. 2024 May 8;18(5):e0012102. doi: 10.1371/journal.pntd.0012102 (PMC11104661; doi:10.1371/journal.pntd.0012102)
Supplement: S1 Table — (DOCX) [file pntd.0012102.s010.docx]

**S1 Table:** Baseline socio-demographic characteristics, water contact information, and history of urinary, genital, and sexual and reproductive health (SRH) signs and symptoms by FGS status diagnosed by colposcopic images (*‘Visual-FGS’)* and molecular methods (*‘Molecular-FGS’)*

|  | | | Visual FGS status  (Hand-held colposcopy) | | | Molecular FGS status  (Genital PCR) | | |
| --- | --- | --- | --- | --- | --- | --- | --- | --- |
| Characteristics | | **Overall^*^**  **(n=950)**  **N (%)** | **Positive**  **(N_tot_=247)**  **N (%)** | **Negative**  **(N_tot_=633)**  **N (%)** | **P-value^**^** | **Positive**  **(N_tot_=68) N(%)** | **Negative**  **(N_tot_=830) N(%)** | **P-value^**^** |
| Socio-behavioural characteristics^+^ | | | | | | | | |
| Age in years | Median (IQR) | 27 (20-38) | 30 (22-41) | 26 (30-36) | 0·005 | 23 (20-30) | 27 (20-38) | 0·01 |
| Marital status | Single | 157 (16·5%) | 47 (19·0%) | 98 (15·5%) | 0·006 | 11 (16·2%) | 136 (16·4%) | 0·95 |
|  | Married or Cohabitating | 659 (69·4%) | 162 (65·6%) | 449 (70·9%) |  | 47 (69·1%) | 579 (69·8%) |  |
|  | Divorced or separated | 82 (8·6%) | 16 (6·5%) | 61 (9·6%) |  | 7 (10·3%) | 71 (8·6%) |  |
|  | Widowed | 51 (5·4%) | 22(8·9%) | 25 (4·0%) |  | 3 (4·4%) | 44 (5·3%) |  |
| Education (highest level) | Any primary school | 500 (52·7%) | 118 (47·8%) | 344 (54·3%) | 0·18 | 40 (58·8%) | 435 (52·4%) | 0·17 |
|  | Any Secondary School | 107 (11·3%) | 22 (8·9%) | 72 (11·4%) |  | 4 (5·9%) | 93 (11·2%) |  |
|  | Training in a Trade | 3 (0·32%) | 1 (0·4%) | 2 (0·32%) |  | 1 (1·5%) | 2 (0·2%) |  |
|  | None | 339 (35·7%) | 106 (42·9%) | 215 (33·9%) |  | 23 (33·8%) | 300 (36·1%) |  |
| Employment status | Working | 9 (0·95%) | 4 (1·6%) | 5 (0·8%) | 0·27 | 1 (1·5%) | 8 (1·0%) | 0·69 |
|  | Not working | 940 (99·1%) | 243 (98·4%) | 628 (99·2%) |  | 67 (98·5%) | 822 (99·0%) |  |
| Frequency of freshwater contact activities | None | 534 (56·3%) | 134 (54·3%) | 361 (57·0%) | 0·16 | 36 (52·9%) | 467 (56·3%) | 0·77 |
|  | At least daily | 365 (38·5%) | 96 (38·9%) | 242 (38·2%) |  | 30 (44·1%) | 318 (38·3%) |  |
|  | At least weekly | 35 (3·7%) | 15 (6·1%) | 18 (2·8%) |  | 1 (1·5%) | 33 (4·0%) |  |
|  | Every 1-2 months | 14 (1·5%) | 2 (0·8%) | 11 (1·7%) |  | 1 (1·5%) | 11 (1·3%) |  |
|  | Every 6-12 months | 1 (0·11%) | 0 | 1 (0·2%) |  | 0 | 1 (0·1%) |  |
| Sexual behaviour characteristics^+^ | | | | | | | | |
| Ever been pregnant | No | 154 (16·2%) | 43 (17·4%) | 95 (15·0%) | 0·56 | 13 (19·1%) | 131 (15·7%) | 0·73 |
|  | Yes | 794 (82·7%) | 204 (82·6%) | 538 (84·8%) |  | 55 (80·9%) | 699 (84·2%) |  |
| Age at sexual debut | Median (IQR) | 17 (15-18) | 17 (15-18) | 17 (15-18) | 0·38 | 16 (15-18) | 17 (16-18) | 0·04 |
| Currently sexually active | Yes | 845 (89·0%) | 204 (82·6%) | 583 (92·1%) | <0·01 | 61 (89·7%) | 738 (88·9%) | 0·84 |
|  | No | 104 (11·0%) | 43 (17·4%) | 50 (7·9%) |  | 7 (10·3%) | 92 (11·1%) |  |
| Previous STI diagnosis | Yes | 85 (9·0%) | 11 (4·5%) | 69 (10·9%) | 0·003 | 5 (7·4%) | 75 (9·0%) | 0·64 |
|  | No | 564 (91·0%) | 236 (95·6%) | 564 (89·1%) |  | 63 (92·7%) | 755 (91·0%) |  |
| Time to get pregnant^+++^  (Overall n=794) | Less than six months | 6 (0·75%) | 3 (1·5%) | 3 (0·7%) | 0·006 | 0 | 6 (0·9%) | 0·88 |
|  | 6-12 months | 10 (1·26%) | 4 (2·0%) | 5 (0·9%) |  | 0 | 8 (1·1%) |  |
|  | More than 1 year | 679 (85·5%) | 175 (85·8%) | 461 (85·9%) |  | 50 (90·9%) | 598 (85·6%) |  |
|  | Unplanned pregnancy | 8 (1·0%) | 5 (2·5%) | 1 (0·2%) |  | 0 | 7 (1·0%) |  |
|  | Refused to answer or do not remember | 91 (11·5%) | 9 (4·4%) | 53 (9·9%) |  | 5 (9·1%) | 80 (11·4%) |  |
| Ultrasound morbidity^++^ | | **Overall^*^**  **(n=800)**  **N (%)** | **Positive**  **(N_tot_=220)**  **N (%)** | **Negative**  **(N_tot_=580)**  **N (%)** | **P-value^**^** | **Positive**  **(N_tot_=60) N(%)** | **Negative**  **(N_tot_=726) N(%)** | **P-value^**^** |
| Bladder wall morbidity | Yes | 9 (1·1%) | 3 (1·4%) | 6 (1·0%) | 0·69 | 2 (3·3%) | 7 (1·0%) | 0·10 |
|  | No | 842 (98·9%) | 217 (98·6%) | 574 (99·0%) |  | 58 (99·7%) | 719 (99·0%) |  |
| Ureter morbidity | Yes | 3 (0·35%) | 0 | 3 (0·5%) | 0·29 | 0 | 3 (0·4%) | 0·62 |
|  | No | 848 (99·7%) | 220 (100%) | 577 (9·5%) |  | 60 (100%) | 723 (99·6%) |  |

^*^ Overall refers to the prevalence of the exposure variable across the study population

^**^ P-value were calculated for the comparison of exposure variables by FGS status.Perason chi-sqared tests were used for comparing categorical variables by FGS status from different diagnostic methods.Wilcoxon-Mann-Whitney was used for comparing continuous variables by FGS status from different diagnostic methods.

^+^The number (N) and corresponding percentage (%) for the socio-demographic and behavioural characteristics were calculated after matching the FGS status with data from the MORBID-FGS questionnaire

^++^The number (N) and corresponding percentage (%) for the ultrasound data were calculated after matching the FGS status dataset with the main MORBID data.

^+++^ Time to get pregnant measures the time it takes for a woman to voluntarily conceive

The percentages are calculated as the proportion of participants with the different characteristics by ‘*visual-FGS’* or ‘*molecular-FGS’* status (i.e. the denominator is the number N from columns)
