# Supplementary material for: Female genital schistosomiasis burden and risk factors in two endemic areas in Malawi nested in the Morbidity Operational Research for Bilharziasis Implementation Decisions (MORBID) cross-sectional study
Source: PLoS Negl Trop Dis. 2024 May 8;18(5):e0012102. doi: 10.1371/journal.pntd.0012102 (PMC11104661; doi:10.1371/journal.pntd.0012102)
Supplement: S2 Table — (DOCX) [file pntd.0012102.s011.docx]

**S2 Table:** Baseline socio-demographic characteristics, water contact information, and history of urinary, genital, and sexual and reproductive health (SRH) signs and symptoms for 950 women across the study population and by the two study districts (Chikwawa and Nsanje) in Southern Malawi.

| Characteristics |  | Overall^*^  (n=950) | Chikwawa (n=388) | Nsanje  (n=562) | p-value^**^ |
| --- | --- | --- | --- | --- | --- |
| Age in years | Median (IQR) | 27 (20-38) | 27 (20-36·5) | 27 (21-38) | 0·96 |
| Socio-behavioural characteristics (N (%) | | | | | |
| Marital status | Single | 157 (16·5%) | 67 (17·3%) | 90 (16·0%) | 0·04 |
|  | Married or Cohabitating | 659 (69·4%) | 277 (71·4%) | 382 (68·1%) |  |
|  | Divorced or separated | 82 (8·6%) | 33(8·5%) | 49 (8·7%) |  |
|  | Widowed | 51 (5·4%) | 11 (2·8%) | 40 (7·1%) |  |
| Education (highest level) | Any primary school | 500 (52·7%) | 229 (59·0%) | 271 (48·3%) | 0·01 |
|  | Any Secondary School | 107 (11·3%) | 36 (9·3%) | 71 (12·7%) |  |
|  | Training in a Trade | 3 (0·32%) | 1 (0·26%) | 2 (0·36%) |  |
|  | None | 339 (35·7%) | 122 (31·4%) | 217 (38·7%) |  |
| Employment status | Working | 9 (0·95%) | 1 (0·26%) | 8 (1·4%) | 0·07 |
|  | Not working | 940 (99·1%) | 387 (99·7%) | 551 (98·6%) |  |
| Frequency of freshwater contact activities | None | 534 (56·3%) | 147 (37·9%) | 387 (69·0%) | <0·001 |
|  | At least daily | 365 (38·5%) | 224 (57·7%) | 141 (25·1%) |  |
|  | At least weekly | 35 (3·7%) | 11 (2·8%) | 24 (4·3%) |  |
|  | Every 1-2 months | 14 (1·5%) | 5 (1·3%) | 9 (1·6%) |  |
|  | Every 6-12 months | 1 (0·11%) | 1 (0·26%) | 0 (0·0) |  |
| Sexual behaviour characteristics | | | | | |
| Ever pregnant | No | 154 (16·2%) | 60 (15·5%) | 94 (16·7%) | 0·61 |
|  | Yes | 794 (82·7%) | 328 (84·5%) | 466 (83·1%) |  |
| Age at sexual debut | Median (IQR) | 17 (15-18) | 16 (15-18) | 17 (16-18) | <0·001 |
| Currently sexually active | Yes | 845 (89·0%) | 368 (94·9%) | 477 (85·0%) | <0·001 |
|  | No | 104 (11·0%) | 20 (5·2 %) | 84 (15·0%) |  |
| Previous STI diagnosis | Yes | 85 (9·0%) | 48 (12·4%) | 37 (6·6%) | 0·002 |
|  | No | 864 (91·0%) | 340 (87·6%) | 524 (93·4%) |  |
| Time to get pregnant  (Overall n=794)^+^ | Less than six months | 6 (0·75%) | 0 | 6 (1·3%) | 0·002 |
|  | 6-12 months | 10 (1·26%) | 3 (0·91%) | 7 (1·5%) |  |
|  | More than 1 year | 679 (85·5%) | 295(90·0%) | 384 (82·3%) |  |
|  | Unplanned pregnancy | 8 (1·0%) | 0 | 8 (1·7%) |  |
|  | Refused to answer or do not remember | 91 (11·5%) | 30 (9·1%) | 61 (13·1%) |  |
| Ultrasound morbidity (Overall N=851) | | | | | |
| Bladder wall morbidity | Yes | 9 (1·1%) | 1 (0·3%) | 8 (1·7%) | 0·05 |
|  | No | 842 (98·9%) | 375 (99·7%) | 467 (98·3%) |  |
| Ureter morbidity | Yes | 3 (0·35%) | 2 (0·53%) | 1 (0·21%) | 0·43 |
|  | No | 848 (99·7%) | 374 (99·5%) | 474 (99·8%) |  |

^*^ Overall prevalence refers to the prevalence of the exposure variable across the study population

^**^ P-value were calculated for the comparison of exposures across Chikwawa and Nsanje districts. Pearson chi-squared tests were used for comparing categorical variables by district.Wilcoxon-Mann-Whitney was used for comparing continuous variables by district.

^+^Time to get pregnant measures the time it takes for a woman to voluntarily conceive.
