# Supplementary material for: Female genital schistosomiasis burden and risk factors in two endemic areas in Malawi nested in the Morbidity Operational Research for Bilharziasis Implementation Decisions (MORBID) cross-sectional study
Source: PLoS Negl Trop Dis. 2024 May 8;18(5):e0012102. doi: 10.1371/journal.pntd.0012102 (PMC11104661; doi:10.1371/journal.pntd.0012102)
Supplement: S3 Table — (DOCX) [file pntd.0012102.s012.docx]

**S3 Table:** Self-reported symptoms across age groups (n=950)

|  | Age groups | | | | | | |
| --- | --- | --- | --- | --- | --- | --- | --- |
|  | | **15-19**  **(N=178)**  **N (%)** | **20-30**  **(N=400)**  **N (%)** | **31-40**  **(N=190)**  **N (%)** | **41-50**  **(N=112)**  **N (%)** | **50+**  **(N=70)**  **N (%)** | **P-value**^*^ |
| Sexual life |  | |  |  |  |  |  |
| Being fearful of pain during sex  (N_tot_=64) | 16 (9·0%) | | 22 (5·5%) | 14 (7·4%) | 8 (7·1%) | 4 (5·7%) | 0·61 |
| Vaginal bleeding after intercourse  (N_tot_=19) | 0 | | 13 (3·3%) | 3 (1·6%) | 3 (2·7%) | 0 | 0·07 |
| Reproductive health |  | |  |  |  |  |  |
| Vaginal itching  (N_tot_=29) | 2 (1·1 %) | | 15 (3·8%) | 7 (3·8%) | 4 (3·6%) | 1 (1·4%) | 0·47 |
| Abdominal pain  (N_tot_=71) | 10 (5·6%) | | 32 (8·0%) | 17 (9·0%) | 7 (6·3%) | 5 (7·1%) | 0·76 |
| Missing menstrual cycle  (N_tot_=247) | 40 (23·8%) | | 121 (32·8%) | 52 (29·7%) | 25 (24·0%) | 9 (13·6%) | 0·009 |
| Vaginal bleeding between periods  (N_tot_=162) | 18 (10·7%) | | 80 (21·7%) | 38 (21·7%) | 14 (13·5%) | 12 (18·2%) | 0·02 |
| Difficulty getting pregnant^+^  (N_tot_=679) | 30 (42·3%) | | 305 (83·8%) | 180 (96·8%) | 100 (95·2%) | 64 (94·1%) | <0·001 |
| Genital sore  (N_tot_=242) | 34 (20·2%) | | 109 (29·5%) | 58 (33·1%) | 23 (22·1%) | 18 (27·3%) | 0·05 |
| Urinary tract |  | |  |  |  |  |  |
| Difficult passing urine  (N_tot_=132) | 27 (15·2%) | | 58 (14·5%) | 24 (12·6%) | 15 (13·4%) | 8 (11·4%) | 0·91 |
| Blood in urine  (N_tot_=33) | 9 (5·1%) | | 16 (4·0%) | 3 (1·6%) | 3 (2·7%) | 2 (2·9%) | 0·41 |

^+^Difficulty getting pregnant is defined as taking more than one year to get pregnant

^*^Pearson Chi-square p-value for the comparison of symptoms across age groups

The percentages are proportion for the number of participants in each age group (i.e. . the denominator is the number N from columns)

The history of signs and symptoms for being fearful of pain during sex, vaginal bleeding after intercourse, vaginal itching, abdominal pain, difficulty passing urine, blood in urine, and difficulty getting pregnant were asked by a study midwife during the MORBID-FGS study.

The history of sign and symptoms for missing menstrual cycle, vaginal bleeding between periods and genital sore were asked in the questionnaire of the main *parent* MORBID study.

A participant could report multiple signs and symptoms. The sum of the values across one column can, therefore, be larger than the total number of observations per age-group.
