# Supplementary material for: Female genital schistosomiasis burden and risk factors in two endemic areas in Malawi nested in the Morbidity Operational Research for Bilharziasis Implementation Decisions (MORBID) cross-sectional study
Source: PLoS Negl Trop Dis. 2024 May 8;18(5):e0012102. doi: 10.1371/journal.pntd.0012102 (PMC11104661; doi:10.1371/journal.pntd.0012102)
Supplement: S4 Table — (DOCX) [file pntd.0012102.s013.docx]

**S4 Table:** Self-reported symptoms across by district

|  | District | | | |
| --- | --- | --- | --- | --- |
| Symptoms | | **Chikwawa**  **N (%)** | **Nsanje**  **N (%)** | **P-value**^*^ |
| Sexual life | |  |  |  |
| Being fearful of pain during sex  (N_tot_=64) | 20 (5·2%) | | 44 (7·8%) | 0·11 |
| Vaginal bleeding after intercourse  (N_tot_=19) | | 5 (1·3%) | 14 (2·5%) | 0·19 |
| Reproductive health |  | |  |  |
| Vaginal itching  (N_tot_=29) | 6 (1·6 %) | | 23 (4·1%) | 0·03 |
| Abdominal pain  (N_tot_=71) | 15 (3·9%) | | 56 (10·0%) | <0·001 |
| Genital sores  (N_tot_=242) | 132 (35·0%) | | 110 (21·8%) | <0·001 |
| Missing menstrual cycle  (N_tot_=247) | 142 (37·7%) | | 105 (20·8%) | <0·001 |
| Difficulty getting pregnant^+^  (N_tot_=679) | 295 (89·9%) | | 384 (82·4%) | 0·003 |
| Vaginal bleeding between periods  (N_tot_=162) | 102 (27·1%) | | 60 (11·9%) | <0·001 |
| Urinary tract |  | |  |  |
| Difficult passing urine  (N_tot_=132) | 35 (9·0%) | | 97 (17·3%) | <0·001 |
| Blood in urine  (N_tot_=33) | 7 (1·8%) | | 26 (4·6%) | 0·02 |

^+^Difficulty getting pregnant is defined as taking more than one year to get pregnant

^*^Pearson Chi-square p-value for the comparison of symptoms across age groups

The percentages are proportion for the number of participants in each age group (i.e. the denominator is the number N from columns)

The history of sign and symptoms for missing menstrual cycle, vaginal bleeding between periods and genital sore were asked in the questionnaire of the main MORBID study. For these variables, the total number of observations in Chikwawa and Nsanje are 377 and 505, respectively.

The history of signs and symptoms for being fearful of pain during sex, vaginal bleeding after intercourse, vaginal itching, abdominal pain, difficulty passing urine, blood in urine, and difficulty getting pregnant (N_tot_=950) were asked by a study midwife during the MORBID-FGS study. For these variables, the total number of observations in Chikwawa and Nsanje are 388 and 562, respectively.
