# Supplementary material for: Female genital schistosomiasis burden and risk factors in two endemic areas in Malawi nested in the Morbidity Operational Research for Bilharziasis Implementation Decisions (MORBID) cross-sectional study
Source: PLoS Negl Trop Dis. 2024 May 8;18(5):e0012102. doi: 10.1371/journal.pntd.0012102 (PMC11104661; doi:10.1371/journal.pntd.0012102)
Supplement: S5 Table — (DOCX) [file pntd.0012102.s014.docx]

**S5 Table:** Self-reported symptoms across the overall study population and by FGS status diagnosed by colposcopy (n=880) and genital PCR (n=899)

|  |  | Visual FGS status  (hand-held colposcopy) | | | Molecular FGS status  (Genital PCR) | | |
| --- | --- | --- | --- | --- | --- | --- | --- |
| Symptoms | **Overall**  **(n=950)**  N (%)^**^ | **Positive**  N (%) | **Negative**  N (%) | **P-value**^**^ | **Positive**  N (%) | **Negative**  N (%) | **P-value**^*^ |
| Sexual life |  |  |  |  |  |  |  |
| Being fearful of pain during sex^+^ | 64 (6·7%) | 17 (6·9%) | 40 (6·3%) | 0·76 | 5 (7·2%) | 53 (6·4%) | 0·75 |
| Vaginal bleeding after intercourse^+^ | 19 (2·0%) | 3 (1·2%) | 13 (2·1%) | 0·40 | 3 (4·3%) | 14 (1·7%) | 0·11 |
| Reproductive health |  |  |  |  |  |  |  |
| Vaginal itching^+^ | 29 (3·1%) | 10 (4·1%) | 16 (2·5%) | 0·23 | 2 (2·9%) | 26 (3·1%) | 0·93 |
| Abdominal pain^+^ | 71 (7·5%) | 19 (7·7%) | 45 (7·1%) | 0·77 | 5 (7·2%) | 62 (7·5%) | 0·97 |
| Amenorrhea^++^ (Missing menstrual cycle) | 247 (26·0%) | 47 (20·4%) | 184 (30·8%) | 0·003 | 18 (27·7%) | 214 (28·5%) | 0·89 |
| Difficulty getting pregnant^(*)+^ | 679 (71·4%) | 175 (68·8%) | 461 (72·8%) | 0·98 | 50 (72·5%) | 598 (72·0%) | 0·27 |
| Genital sores^++^ | 242 (25·5%) | 53 (22·9%) | 169 (28·3%) | 0·12 | 21 (32·3%) | 206 (27·4%) | 0·40 |
| Vaginal bleeding between periods^++^ | 162 (17·1%) | 31 (13·4%) | 121 (20·3%) | 0·20 | 14 (21·5%) | 142 (18·9%) | 0·61 |
| Urinary tract |  |  |  |  |  |  |  |
| Difficult passing urine^+^ | 132 (13·9%) | 37 (15·0%) | 86 (13·6%) | 0·59 | 15 (21·7%) | 112 (13·5%) | 0·06 |
| Blood in urine^+^ | 33 (3·4%) | 8 (3·2%) | 23 (3·6%) | 0·78 | 7 (10·1%) | 24 (2·9%) | 0·001 |

^(*)^ Difficulty getting pregnant is defined as taking more than one year to get pregnant.

^*^Overall refers to the total prevalence of different signs and symptoms across the study population.

^**^Pearson Chi-square p-value for the comparison of symptoms across FGS status.

The percentages are proportion for the number of participants by FGS status (i.e. the denominator is the number N from columns)

^+^The number (N) and corresponding percentage (%) for the symptoms by *‘visual-FGS’* and *‘molecular-FGS’* status were calculated after matching the corresponding FGS status dataset with the MORBID-FGS questionnaire data. For these variables, the total number of observations available on *‘visual-*FGS*’* was 880, 247 positive and 633 negatives. The total number of observations available after matching with *‘molecular-FGS*’ status was 899, 69 positive and 831 negatives.

^++^ The number (N) and corresponding percentage (%) for the symptoms by *‘visual-FGS’* and *‘molecular-FGS’* status were calculated after matching the corresponding FGS st atus dataset with the main MORBID questionnaire data. For these variables, the total number of observations available on *‘visual-*FGS*’* was 828, 231 positive and 597 negatives. The total number of observations available after matching with *‘molecular-FGS*’ status was 816, 65 positive and 751 negatives.
