# Supplementary material for: Female genital schistosomiasis burden and risk factors in two endemic areas in Malawi nested in the Morbidity Operational Research for Bilharziasis Implementation Decisions (MORBID) cross-sectional study
Source: PLoS Negl Trop Dis. 2024 May 8;18(5):e0012102. doi: 10.1371/journal.pntd.0012102 (PMC11104661; doi:10.1371/journal.pntd.0012102)
Supplement: S6 Table — (DOCX) [file pntd.0012102.s015.docx]

**S6 Table:** Self-reported symptoms by FGS typical cervicovaginal lesions detected using the EVA MobileODT colposcope

|  | Homogeneous yellow sandy patches | | | Grainy sandy patches | | | Abnormal blood vessels | | | Rubbery papules | | |
| --- | --- | --- | --- | --- | --- | --- | --- | --- | --- | --- | --- | --- |
|  | **Positive**  (N=172)  N (%) | **Negative**  (N=569)  N (%) | **p-value^**^** | **Positive**  (N=23)  N (%) | **Negative**  (N=718)  N (%) | **P-value^**^** | **Positive**  (N=13)  N (%) | **Negative**  (N=728)  N (%) | **P-value^**^** | **Positive**  (N=6)  N (%) | **Negative**  (N=735)  N (%) | **P-value^**^** |
| Sexual life |  |  |  |  |  |  |  |  |  |  |  |  |
| Being fearful of pain during sex | 16 (9·3%) | 41 (7·2%) | 0·40 | 1 (4·3%) | 56 (7·8%) | 0·45 | 2 (15·4%) | 55 (7·6%) | 0·28 | 1 (16·7%) | 56 (7·6%) | 0·40 |
| Vaginal bleeding after intercourse | 3 (1·7%) | 13 (2·3%) | 0·65 | 0 | 16 (2·2%) | 0·44 | 0 | 16 (2·2%) | 0·60 | 0 | 16 (2·2%) | 0·72 |
| Reproductive health |  |  |  |  |  |  |  |  |  |  |  |  |
| Vaginal itching | 10 (5·8%) | 16 (2·8%) | 0·07 | 1 (4·3%) | 25 (3·5%) | 0·93 | 0 | 26 (3·6%) | 0·50 | 0 | 26 (3·5%) | 0·64 |
| Abdominal pain | 17 (9·9%) | 47 (8·3%) | 0·55 | 1 (4·3%) | 63 (8·8%) | 0·38 | 3 (23·1%) | 61 (8·4%) | 0·06 | 1 (16·7%) | 63 (8·6%) | 0·47 |
| Missing menstrual cycle | 41 (23·8%) | 190 (33·4%) | 0·02 | 2 (8·7%) | 229 (31·9%) | 0·01 | 3 (23·1%) | 228 (31·3%) | 0·49 | 1 (16·7%) | 230 (31·3%) | 0·42 |
| Difficulty getting pregnant ^+^ | 147 (85·5%) | 489(85·9%) | 0·88 | 17 (73·9%) | 619 (86·2%) | 0·10 | 12 (92·3%) | 624 (85·7%) | 0·50 | 4 (66·7%) | 632 (86·0%) | 0·18 |
| Genital sores | 45 (26·2%) | 177 (31·1%) | 0·19 | 5 (21·7%) | 217 (30·2%) | 0·28 | 1(7·7%) | 221 (30·4%) | 0·08 | 0 | 222 (30·2%) | 0·11 |
| Vaginal bleeding between periods | 26 (15·1%) | 126 (22·1%) | 0·04 | 0 | 152 (21·2%) | 0·01 | 3 (23·1%) | 149 (20·5%) | 0·87 | 0 | 152 (20·7%) | 0·21 |
| Urinary tract |  |  |  |  |  |  |  |  |  |  |  |  |
| Difficult passing urine | 33 (19·2%) | 90 (15·8%) | 0·35 | 2 (8·7%) | 121 (16·9%) | 0·22 | 2 (15·4%) | 121 (16·6%) | 0·94 | 1 (16·7%) | 122 (16·6%) | 0·98 |
| Blood in urine | 5(2·9%) | 26 (4·6%) | 0·32 | 1 (4·3%) | 30 (4·2%) | 0·93 | 1 (7·7%) | 30 (4·1%) | 0·51 | 1 (16·7%) | 30 (4·1%) | 0·12 |

^+^Difficulty getting pregnant is defined as taking more than one year to get pregnant.

^**^Pearson Chi-square p-value for the comparison of symptoms across FGS status.

The percentages are calculated as the proportion of participants showing symptoms by lesion observed using colposcopy (i.e. the denominator is the number N from columns)

The total number (N) reflect the number of observations available after
