# Supplementary material for: Female genital schistosomiasis burden and risk factors in two endemic areas in Malawi nested in the Morbidity Operational Research for Bilharziasis Implementation Decisions (MORBID) cross-sectional study
Source: PLoS Negl Trop Dis. 2024 May 8;18(5):e0012102. doi: 10.1371/journal.pntd.0012102 (PMC11104661; doi:10.1371/journal.pntd.0012102)
Supplement: S7 Table — (DOCX) [file pntd.0012102.s016.docx]

**S7 Table:** Distribution of *‘visual-FGS’* (cervical lesion detected by EVA MobileODT) positives (N=241) by *‘molecular-FGS’* status (parasite DNA detection by PCR)

| Cervical lesions observed in ‘*visual-FGS’* positives.  (N=237)^*^ | *‘Molecular-FGS’* positive  (N=33)  N (%) | *‘Molecular-FGS’* negative  (N=204)  N (%) | P-value^**^ |
| --- | --- | --- | --- |
| Homogeneous yellow sandy patches  (N=198) ^*^ | 18 (54·6%) | 180 (88·3%) | 0·45 |
| Grainy sandy patches  (N=28) ^*^ | 3 (9·1%) | 25 (12·3%) | 0·56 |
| Rubbery papules  (N=7) ^*^ | 1 (3·0%) | 6 (2·9%) | 0·52 |
| Abnormal blood vessels  (N=15) ^*^ | 1 (3·0%) | 14 (6·9%) | 0·87 |

^*^The total numbers (N) reflect the number of observations available after matching the *‘visual-FGS’* and *‘molecular-FGS’* datasets

^**^Pearson Chi-square p-value for the comparison of symptoms across FGS status

The percentages were calculated as the proportion of participants with the different cervical lesions by molecular FGS status ((i.e. the denominator is the number N from columns)
