# Supplementary material for: Female genital schistosomiasis burden and risk factors in two endemic areas in Malawi nested in the Morbidity Operational Research for Bilharziasis Implementation Decisions (MORBID) cross-sectional study
Source: PLoS Negl Trop Dis. 2024 May 8;18(5):e0012102. doi: 10.1371/journal.pntd.0012102 (PMC11104661; doi:10.1371/journal.pntd.0012102)
Supplement: S8 Table — (DOCX) [file pntd.0012102.s017.docx]

**S8 Table:** Distribution of *‘visual-FGS’* by EVA MobileODT colposcopy and *‘molecular-FGS’* by PCR of genital swabs across age groups

|  | 15-19 years  N (%) | 20-30 years  N (%) | 31-40 years  N (%) | 41-50 years  N (%) | 50+ years  N (%) | P-value^**^ |
| --- | --- | --- | --- | --- | --- | --- |
| *‘Visual-FGS’* positive (EVA MobileODT colposcopy)  (N = 247)^*^ | 42 (25·6%) | 87 (23·7%) | 56 (31·5%) | 30 (28·8%) | 32 (47·8%) | 0·001 |
| *‘Molecular-FGS’* positive (PCR on genital swab)  (N= 68)^*^ | 15 (9·0 %) | 37 (9·5%) | 9 (5·0%) | 2 (1·9%) | 5 (7·7%) | 0·05 |
| *S· haematobium* positive  (Urine filtration)  (N=38) | 11 (7·2%) | 15 (6·7%) | 8 (6·6%) | 2 (3·7%) | 2 (6·1%) | 0·93 |

^*^ The total number (N) reflect the number of observations available after matching the *‘visual-FGS’, ‘molecular-FGS’* with the main MORBID-FGS dataset

^**^Pearson Chi-squared p-value for the comparison of FGS status across age-groups
