# Supplementary material for: Female genital schistosomiasis burden and risk factors in two endemic areas in Malawi nested in the Morbidity Operational Research for Bilharziasis Implementation Decisions (MORBID) cross-sectional study
Source: PLoS Negl Trop Dis. 2024 May 8;18(5):e0012102. doi: 10.1371/journal.pntd.0012102 (PMC11104661; doi:10.1371/journal.pntd.0012102)
Supplement: S9 Table — (DOCX) [file pntd.0012102.s018.docx]

**S9 Table:** FGS typical cervical lesions observed using the Smart-scope colposcope

| Cervical lesions observed by periwinkle colposcopy | N (%)  (N_tot_=24) |
| --- | --- |
| Homogeneous yellow sandy patches | 10 (41·7%) |
| Grainy sandy patches | 9 (37·5%) |
| Rubbery papules | 0 (0%) |
| Abnormal blood vessels | 8 (33·3%) |
