# Supplementary material for: Female genital schistosomiasis burden and risk factors in two endemic areas in Malawi nested in the Morbidity Operational Research for Bilharziasis Implementation Decisions (MORBID) cross-sectional study
Source: PLoS Negl Trop Dis. 2024 May 8;18(5):e0012102. doi: 10.1371/journal.pntd.0012102 (PMC11104661; doi:10.1371/journal.pntd.0012102)
Supplement: S10 Table — (DOCX) [file pntd.0012102.s019.docx]

**S10 Table.** Generalized linear mixed models (GLMM) parameter estimates for association between ‘*molecular-FGS’* and *S. haematobium* infection status by urine filtration (n=537)

| Characteristic | AOR*^1^* | 95% CI*^1^* | p-value |
| --- | --- | --- | --- |
| *S. haematobium* by urine microscopy | 7·5 | 3·3-17·2 | <0·001 |
| *Standardized age* | 0·8 | 0·6- 1·2 | 0·3 |
| *^1^* AOR = Adjusted Odds Ratio, CI = Confidence Interval  Model adjusted for standardized age.  Standardized age was selected as a confounder based on univariable associations with outcomes and *priori* knowledge on the distinct age-related differences in FGS morbidity manifestations.  Village was treated as a random effect. | | | |
