# Supplementary material for: Female genital schistosomiasis burden and risk factors in two endemic areas in Malawi nested in the Morbidity Operational Research for Bilharziasis Implementation Decisions (MORBID) cross-sectional study
Source: PLoS Negl Trop Dis. 2024 May 8;18(5):e0012102. doi: 10.1371/journal.pntd.0012102 (PMC11104661; doi:10.1371/journal.pntd.0012102)
Supplement: S11 Table — (DOCX) [file pntd.0012102.s020.docx]

**S11 Table.** Generalized linear mixed models (GLMM) parameter estimates for association between ‘*visual-FGS’* status by EVA Mobile ODT and *S. haematobium* infection by urine filtration (n=544)

| **Characteristic** | **AOR***^1^* | **95% CI***^1^* | **p-value** |
| --- | --- | --- | --- |
| *S· haematobium* by microscopy | 1·4 | 0·6-3·2 | 0·5 |
| *Standardized age* | 1·4 | 1·2- 1·8 | 0·001 |
| *^1^* AOR = Adjusted Odds Ratio, CI = Confidence Interval  Model adjusted for standardized age.  Standardized age was selected as a confounder based on univariable associations with outcomes and *priori* knowledge on the distinct age-related differences in FGS morbidity manifestations.  Village was treated as a random effect. | | | |
